# Factors associated with mental health consultation in South Korea

**DOI:** 10.1186/s12888-018-1592-3

**Published:** 2018-01-22

**Authors:** Jieun Jang, Sang Ah Lee, Woorim Kim, Young Choi, Eun-Cheol Park

**Affiliations:** 1 Department of Public Health, Graduate School, Yonsei University, Seoul, Republic of Korea; 2 Institute of Health Services Research, Yonsei University, Seoul, Republic of Korea; 3 Department of Preventive Medicine & Institute of Health Services Research, Yonsei University College of Medicine, 50 Yonsei-ro, Seodaemun-gu, Seoul, 120-752 Republic of Korea

**Keywords:** Depression, Mental health consultation, Education, Depress, Mental health

## Abstract

**Background:**

The aim of this study was to examine factors associated with the use of mental health consultation for depressive symptoms.

**Methods:**

We used data from the 2013 Community Health Survey, which included responses from 13,269 individuals who reported that they had experienced depressive symptoms for more than 2 weeks in Korea. We investigated associations between mental health consultation rates for depressive symptoms and sociodemographic, socioeconomic, and health-related factors. Logistic regression analysis was used to examine the significance of associations.

**Results:**

Among participants who report depressive symptoms, 16.0% (*n* = 2120) undergo mental health consultation. Respondents with a college education or over are more likely to undergo mental health consultation (odds ratio (OR) = 1.49; 95% CI: 1.21–1.84) than respondents with less education. Individuals aged 70 years or above are less likely to receive mental health consultation than those aged between 19 and 29 years. Females exhibit higher mental health consultation rates than males. Respondents who are divorced show greater odds of receiving mental health consultation than respondents who are married and cohabitate with their spouse.

**Conclusions:**

This study indicates that rates of use of mental health consultation services are lower among older adults and men and higher among divorced people. Educational level shows a significant positive association with mental health consultation among Koreans. The results could have implications for mental health policy in many ways in Korea.

## Background

Questions about why individuals do not seek mental health services for treatment of their mental illness and how to improve use of mental health consultation services are important topics worldwide [1, 2]. Depression is a common disease, affecting at least 300 million people around world [3]. However, people with depression often still do not receive mental health consultation for depression [4]. People with depression are less likely to obtain continuous treatment for depression due to lack of information on how to manage depression and the stigma associated with the condition [5–7]. The World Health Organization projected for the year 2030 that depression would be a major cause of the disease burden [8]. Depression is a significant public health problem [9].

Depression is an important issue, because it is a major cause of suicide [10]. Mounting evidence suggests that depressive disorder is strongly associated with completed suicide [11, 12]. About 60% of all suicides are estimated to stem from depression and other mood disorders [13–16]. Organisation for Economic Co-operation and Development (OECD) statistics indicate that, in 2013 and 2014, Korea ranked first in suicide rates among OECD countries [17]. Therefore, addressing depression is an important issue for Korea.

Mental health consultation is an effective treatment for depressive symptoms, but many do not seek the help of mental health services [18–20]. The results of the 2013 Community Health Survey (CHS) indicate that approximately 5.8% of Koreans are affected by depressive symptoms, but only 16% of those depressed receive mental health consultation for their depression symptoms. Mental health consultation for treatment of depression is as important as the use of medications [18]. Despite the fact that mental health consultation is an effective treatment for depression, it is a real problem that people with depressive symptoms do not receive a mental health consultation.

Utilization of mental health services has been found to be related to several factors [18, 19]. Howard et al. noted that mental health services were associated with educational level, marital status, and race [21]. Vessey, John T et al. reported that the most educated people are the most likely to utilize mental health services [22]. Moreover, divorced people are the most likely to consult with mental health services than married, bereaved, and unmarried people [22]. They identified that there was no strong relationship between the utilization of mental health services and income level [22]. In addition, stigma is a barrier to the use of mental health consultation. Stigma has been shown to influence help seeking and consistency with treatment for mental illness [23]. People with suicidal thoughts also may have more severe depressive symptoms than people with no suicidal thoughts. Severe mental disorder is significantly related to the use of mental health services [24]. Demyttenaere K. et al. indicated that people with serious mental disorders show much higher use of mental health treatment than people with moderate mental disorders [24].

Seeking help for mental health problems has been shown to be related with several factors. These include age, gender, and educational level [4, 21]. Educational level, in particular, exhibits several associations with the use of mental health consultation. A higher educational level appears to dispose an individual to greater access to health information [25, 26]. Moreover, individuals who have attained higher educational levels are more likely to actively respond to the information they receive [25, 26]. Meanwhile, negative attitudes towards mental health services and distrust of the effect of treatment also affect the utilization of mental health consultation services [27]. Therefore, education may be essential in improving help-seeking attitudes and willingness to undergo mental health consultation.

The purpose of this study was to identify several factors related to the use of mental health consultation. In Korea, the government supports mental health promotion centers in each city [28]. Since 2005, a mental care health hotline (+82-129) has provided mental health consultations from mental health experts anywhere and anytime [29]. Additionally, the government has proposed policies to address depression rates and recommend mental health consultation for depressive symptoms in Korea. Therefore, it is essential to study factors associated with the use of mental health consultation.

## Methods

### Study population

Data from the nationwide 2013 Community Health Survey (CHS) were used for this study. The CHS used a cross-sectional study design and was performed in cooperation with the Korea Centers for Disease Control and Prevention and municipalities in Korea. The Korea Centers for Disease Control and Prevention approved the use of data from CHS through the CHS website (https://chs.cdc.go.kr/chs/index.do). A total of 228,781 individuals responded to the survey. Our study population consisted only of those who had depressive symptoms. We selected participants using the question ‘Within the last year, did you experience any emotions, such as sadness or despair, continuously for more than 2 weeks, which increased difficulties in daily life?’ Respondents who answered ‘no’ (*n* = 215,345) and didn’t answer the question (*n* = 59) were excluded from our study population. Respondents who answered ‘yes’ were classified as individuals with depressive symptoms (*n* = 13,377). We excluded individuals with data missing for use of mental health consultation services (*n* = 1), marital status (*n* = 11), educational level (*n* = 26), occupation (*n* = 11), stress level (*n* = 16), smoking status (*n* = 3), suicidal thoughts (*n* = 11), perceived health status (*n* = 2), and physical activity (*n* = 27). Therefore, a final sample population of 13,269 people was included for analysis in this study.

### Variables

The dependent variable was participants’ use of mental health consultation services. Participants who had depressive symptoms (*n* = 13,269) were asked the question, ‘Did you receive any mental health consultation (e.g., medical institution, professional consulting institution, local health centre) for these symptoms?’ We examined the participants’ use of mental health consultation services based on a ‘yes’ or ‘no’ answer.

The main independent variable of interest in this study was education level of the participants. We examined education attainment using a survey question. The question asked for the respondents to indicate the last school grade that was completed. In order to compare the educational level from a global perspective, the International Standard Classification of Education (ISCED) was used in the analysis. International Standard Classification is one of the standard frameworks to compare education statistics. Low educational level included ISCED classification of 0–1 (early childhood education and primary education) and medium educational level included ISCED classification of 2 (lower secondary education). High education was defined as ISCED classification of 3 (upper secondary education), and college or over as ISCED classification of 5 to 8 (short-cycle tertiary education, bachelor or equivalent, master or equivalent, doctoral or equivalent.

Other variables in the analysis included sociodemographic, socioeconomic, health-behaviour, and health-condition variables. The sociodemographic factors were age (19 to 29 years, 30 to 39 years, 40 to 49 years, 50 to 59 years, 60 to 69 years, 70 or above) and gender (male, female). The socioeconomic factors were marital status (married and cohabit, married and non-cohabit, bereaved, divorced, unmarried), region (capital, urban, rural), household income level (divided into quartiles), and occupation (managers, professionals, clerical support workers, service workers, sales worker, skilled agricultural, forestry and fishery workers, craft and related trades workers, plant and machine operators, and assemblers, elementary occupations, armed forces occupations, others).

The capital region included Seoul, which is the capital of Korea, and Gyeonggi province, which surrounds Seoul. About a population of 20 million (about 50% of Korea) live in the capital region. Urban areas included metropolitan cities usually with a population of about 1 million. Rural areas included the remaining geographic areas, excluding capital and urban regions. The standards of dividing house income level per year were less than 8,400,000 won (about 75,350,000 USD), less than 24,000,000 won (about 215,290,000 USD), less than 40,000,000 won (about 358,800,000 USD), and more than 40,000,000 or above (about 358,800,000 USD). Occupation was classified by the Korean Standard Classification of Occupations (KSCO). The KSCO is based on the International Standard Classification of Occupations. The International Standard Classification of Occupations is one of the standard frameworks for classifying jobs according to the tasks and duties undertaken in the job [30]. This classification was simplified as high-skilled white collar, low-skilled white collar, high-skilled blue collar, low-skilled blue collar, and others. The high-skilled white collar group included managers, professionals, and clerical support workers. Low-skilled white collar workers included service workers and sales workers. The high-skilled blue collar group included skilled agricultural, forestry, and fishery workers, as well as craft and related trade workers. Low-skilled blue collar workers included plant and machine operators and assemblers and elementary occupations. Others included armed forces occupations, students, and other job statuses.

Health-behaviour factors included physical activity (inactive and active by metabolic equivalent task), smoking status (never or former, current [within a year]) and alcohol consumption (never or former, current [within a year]). Health-condition factors included stress level (very, little no), suicidal thoughts (yes or no), sleep duration (<7 h, 7 to 8 h, ≥9 h), and perceived health status (good, normal, bad). Participants were asked the question, ‘Did you have suicidal thoughts within the last year?’ And we examined the participants’ suicidal thoughts based on a ‘yes’ or ‘no’ answer.

### Statistical analysis

Statistical analysis was performed using SAS software, version 9.4 (SAS Institute, Cary, NC, USA). All analyses included the use of weighted variables. Chi-square tests were used to confirm the significant differences between respondents who did and did not obtain mental health consultation for depression symptoms. Logistic regression analysis was used to determine odds ratios (ORs) and 95% confidence intervals (CIs). Subgroup analysis was performed according to age and suicidal thoughts. A *p*-value <0.05 was considered to indicate a statistically significant result.

## Results

Table 1 presents the general characteristics of the study populations. There are 4019 males (30.3%) and 9250 females (69.7%) included in this study. Of the 13,269 participants who have depressive symptoms, 2120 (16.0%) received mental health consultation and 11,149 (84.0%) did not. The rate of use of mental health consultation is the lowest among participants with low educational level (14.6%) relative to among college or over (15.5%). Of the 13,269 participants, the rate of receiving mental health consultation is the lowest among the 70 years or above participants (12.4%). The rate of use of mental health consultation is the highest among divorced people (21.8%). Moreover, females (17.3%) show higher mental health consultation rates than males (13.0%).

**Table 1:** General characteristics of the study population

Variables	Total		Mental health consultation				*P*-value*
			YES		NO		
	N	%	N	%	N	%	
Total	13,269	100.0	2120	16.0	11,149	84.0	
Educational level - ISCED							0.0005
Low	4832	36.4	704	14.6	4128	85.4	
Medium	1606	12.1	295	18.4	1311	81.6	
High	4119	31.0	700	17.0	3419	83.0	
College or over	2712	20.4	421	15.5	2291	84.5	
Age							<.0001
19–29	1305	9.8	210	16.1	1095	83.9	
30–39	1745	13.2	292	16.7	1453	83.3	
40–49	2144	16.2	365	17.0	1779	83.0	
50–59	2695	20.3	453	16.8	2242	83.2	
60–69	2259	17.0	412	18.2	1847	81.8	
70≤	3121	23.5	388	12.4	2733	87.6	
Gender							<.0001
Male	4019	30.3	523	13.0	3496	87.0	
Female	9250	69.7	1597	17.3	7653	82.7	
Household income level							0.0129
Quartile 1 (lowest)	3518	26.5	536	15.2	2982	84.8	
Quartile 2	3349	25.2	595	17.8	2754	82.2	
Quartile 3	3048	23.0	468	15.4	2580	84.6	
Quartile 4 (highest)	3354	25.3	521	15.5	2833	84.5	
Region							0.7874
Capital (Seoul, Gyeonggi)	4366	32.9	705	16.1	3661	83.9	
Urban	2604	19.6	423	16.2	2181	83.8	
Rural area	6299	47.5	992	15.7	5307	84.3	
Marital status							<.0001
Married-cohabit	7638	57.6	1178	15.4	6460	84.6	
Married-no cohabit	449	3.4	72	16.0	377	84.0	
Bereaved	2440	18.4	364	14.9	2076	85.1	
Divorced	908	6.8	198	21.8	710	78.2	
Unmarried	1834	13.8	308	16.8	1526	83.2	
Occupation							<.0001
High-skilled white collar	1756	13.2	215	12.2	1541	87.8	
Low-skilled white collar	1499	11.3	234	15.6	1265	84.4	
High-skilled blue collar	1528	11.5	194	12.7	1334	87.3	
Low-skilled blue collar	1469	11.1	193	13.1	1276	86.9	
Others	7017	52.9	1284	18.3	5733	81.7	
Physical activity							0.0265
Inactive	8932	67.3	1471	16.5	7461	83.5	
Active	4337	32.7	649	15.0	3688	85.0	
Smoking status							0.3816
Never or former	10,807	81.4	1741	16.1	9066	83.9	
Current	2462	18.6	379	15.4	2083	84.6	
Alcohol consumption							0.0023
Never or former	5289	39.9	908	17.2	4381	82.8	
Current (within a year)	7980	60.1	1212	15.2	6768	84.8	
Stress level							<.0001
Very	8441	63.6	1590	18.8	6851	81.2	
Little	4114	31.0	451	11.0	3663	89.0	
No	714	5.4	79	11.1	635	88.9	
Suicidal Thought							<.0001
Yes	7295	55.0	1472	20.2	5823	79.8	
No	5974	45.0	648	10.8	5326	89.2	
Sleeping duration							0.1173
Less than 7 h	7400	55.8	1193	16.1	6207	83.9	
7–8 h	5162	38.9	797	15.4	4365	84.6	
9 h or over	707	5.3	130	18.4	577	81.6	
Perceived health status							<.0001
Good	2488	18.8	262	10.5	2226	89.5	
Normal	4810	36.2	687	14.3	4123	85.7	
Bad	5971	45.0	1171	19.6	4800	80.4	

* *P*-values calculated by the chi-square test

Table 2 presents the results for the factors associated with the use of mental health consultation services for depressive symptoms. Educational level shows a significant positive association with the use of mental health consultation. Respondents in the college or over level are more likely to receive mental health consultation for depressive symptoms (OR = 1.49; 95% CI: 1.21–1.84), compared to respondents in the low educational level. Individuals in the 70 years or above age group are less likely to receive mental health consultation (OR = 0.46; 95% CI: 0.33–0.64) than the 19 to 29 years old groups. Females shows higher mental health consultation rates (OR = 1.19; 95% CI: 1.02–1.38) than males. There is no significant association between household income level and utilization of mental health consultation. Respondents who are divorced have greater odds of receiving mental health consultation (OR = 1.32; 95% CI: 1.08–1.62) than respondents who are married and cohabitate with their spouse.

**Table 2:** Factors associated with mental health consultation ^a^

Variables	Mental health consultation	
	Adjusted OR	95%CI
Educational level - ISCED		
Low	1.00	–
Medium	1.32	(1.09–1.60)
High	1.35	(1.12–1.63)
College or over	1.49	(1.21–1.84)
Age		
19–29	1.00	–
30–39	0.89	(0.71–1.13)
40–49	0.88	(0.69–1.13)
50–59	0.80	(0.61–1.04)
60–69	0.86	(0.64–1.17)
70≤	0.46	(0.33–0.64)
Gender		
Male	1.00	–
Female	1.19	(1.02–1.38)
Household income level		
Quartile 1 (lowest)	1.00	–
Quartile 2	1.12	(0.96–1.31)
Quartile 3	1.16	(0.97–1.37)
Quartile 4 (highest)	1.17	(0.98–1.41)
Region		
Capital(Seoul, Gyeonggi)	1.00	–
Urban	0.98	(0.85–1.14)
Rural area	0.99	(0.88–1.12)
Marital status		
Married-cohabit	1.00	–
Married-no cohabit	1.29	(0.95–1.73)
Bereaved	1.23	(1.04–1.47)
Divorced	1.32	(1.08–1.62)
Unmarried	1.12	(0.91–1.38)
Occupation		
High-skilled white collar	1.00	–
Low-skilled white collar	1.08	(0.86–1.35)
High-skilled blue collar	0.92	(0.66–1.27)
Low-skilled blue collar	1.00	(0.78–1.27)
Others	1.54	(1.28–1.86)
Physical activity		
Inactive	1.00	–
Active	1.13	(0.99–1.28)
Smoking status		
Never or former	1.00	–
Current	0.98	(0.83–1.15)
Alcohol consumption		
Never or former	1.00	–
Current (within a year)	0.81	(0.71–0.93)
Stress level		
Very	1.00	–
Little	0.65	(0.57–0.75)
No	0.78	(0.58–1.07)
Suicidal Thought		
Yes	2.15	(1.89–2.43)
No	1.00	–
Sleeping duration		
Less than 7 h	1.00	–
7–8 h	1.07	(0.95–1.20)
9 h or over	1.13	(0.91–1.41)
Perceived health status		
Good	1.00	–
Normal	1.31	(1.11–1.55)
Bad	1.89	(1.58–2.26)

^a^ Logistic regression analysis was used to determine odds ratios (ORs) and 95% confidence intervals (CIs). A total of 13,269 participants were included in the analysis. An example of how to interpret this figure is as follows: ‘respondents who received a college or over education are more likely to receive mental health consultation for depressive symptoms (OR = 1.49; 95% CI: 1.21–1.84), compared with respondents with low educational level’

**Fig. 1:**
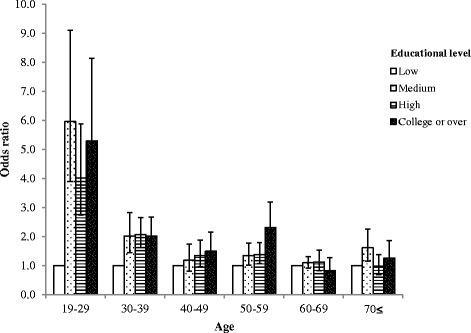
Subgroup analysis of mental health consultation according to educational level stratified by age. An example of how to interpret this figure is as follows: ‘compared with individuals 70 years or above in age, respondents in the 19 to 29 years old groups with college or over educational level have a greater odds ratio for mental health consultation.’ Control variables include gender, region, house income level, marital status, occupation, physical activity, smoking status, alcohol consumption, stress level, suicidal thoughts, sleeping duration, and perceived health status

**Fig. 2:**
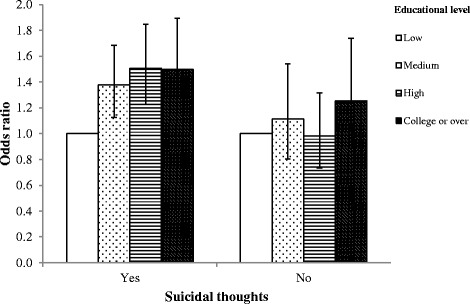
Subgroup analysis of mental health consultation according to educational level stratified by suicidal thoughts. An example of how to interpret this figure is as follows: ‘people who have suicidal thoughts have a stronger association between educational level and use of mental health consultation.’ Control variables include age, gender, region, house income level, marital status, occupation, physical activity, smoking status, alcohol consumption, stress level, sleeping duration, and perceived health status

Figure 1 presents the results for the subgroup analysis of the association between educational level and use of mental health consultation stratified by age. The analysis reveal that, compared to those 70 years or above in age, respondents in the 19 to 29 years old groups in the college or over educational level have a greater odds ratio for mental health consultation. Figure 2 presents the results for the subgroup analysis of the association between educational level and use of mental health consultation stratified according to suicidal thoughts. People who have suicidal thoughts have a stronger association between educational level and use of mental health consultation.

## Discussion

The aim of this study is to identify factors associated with the use of mental health consultation in Korea. The findings reveal that educational level has a positive association with the use of mental health consultation services. Individuals aged 70 years or above tend to undergo mental health consultation less than those aged between 19 and 29 years. Females undergo more mental health consultations than males. Divorced people undergo the most mental health consultation, compared to respondents who are married and cohabitate with their spouse. There is no significant association between household income level and utilization of mental health consultation. Overall, younger people tend to show stronger correlation between educational level and the use of mental health consultation. People who have suicidal thoughts show a stronger association between educational level and use of mental health consultation.

In the present study, we note a significantly positive association between educational levels and the utilization of mental health services. This association may be explained by several reasons. First, highly educated people are more likely to have active attitudes toward mental health services. Individuals who achieve higher educational levels are better at managing their lives, compared to individuals with lower levels of education [31, 32]. The process of studying increases self-confidence and motivation [31]. These characteristics are essential for successful problem-solving, and highly-educated people tend to solve their difficulties using an active attitude approach [31–33]. Second, less educated people tend not to recognize their mental health problems [22]. Mental health services are a type of help-seeking process [21]. These help-seeking processes consist of several steps, including recognizing the problem, realizing the necessary of help, and contacting health services [21]. Because educational level influences awareness of a health care problem, use of mental health services may differ according to the level of education. Third, highly educated people also have more knowledge about mental health services [34, 35]. They have more opportunities to obtain information about how to manage depression (e.g., receiving a mental health consultation) and access to mental health services [25, 26]. Finally, education may influence stigma about mental health services. Stigma of what others could think may be a common barrier for contacting mental health services [21]. The stigma associated with mental health consultation influences delays in seeking assistance [36]. Previous studies showed that highly educated people have less stigma about mental health services [37, 38]. Koreans with mental health problems may have more prejudice against mental health problem than normal people [39]. Also, they tend to avoid making their mental health problems known to other people [39]. Jang et al. identified that such stigma may be influenced by the cultural background in Korea [40].

The present study shows that gender and marital status are associated with the utilization of mental health services, while income level has no significantly associations. The results of this study indicated that older adults were less likely to receive mental health consultation. This result was similar to the results of previous studies. Older adults may be less aware of a mental health problem, have a higher sense of self-sufficiency, and be more conscious of the disgrace associated with receipt of mental health services [41, 42]. Females are more likely to use mental health services than males. This correspond with previous studies [22, 43]. Females may be more likely to recognize their emotional problems than men who had similar symptoms [44]. Moreover, females tend to share their emotion with others and be more openness to acknowledging mental health problems [4, 22]. Divorced people are more likely to have mental health services than people who are married and cohabit in the study. In many cases, people seek consultation to receive advice for marital problems before divorce [45]. Also, pre-divorce counseling could influence access to mental health consultation. John. T. et al. also identified that separated or divorced people were the most likely to seek mental health services, and our study correspond with previous studies [22].

In the age group of individuals between 19 and 29 years, the association between educational level and use of mental health consultation is stronger than adults 70 years or older. Overall, younger people tend to show stronger correlation between educational level and the use of mental health consultation. This may be related to several factors. Older adults are more prejudiced about the use of mental health consultation and less aware of the severity of their depressive symptoms [41, 42]. Also, the fact that education level influences young people more could be implicated in developing mental health policies.

People who have suicidal thoughts show a stronger association between educational level and use of mental health consultation. People with suicidal thoughts have positive association between educational level and high rates of use of mental health consultation. Therefore, it is necessary to strengthen mental health policies in educational aspects, such as educating people how to manage the depressive symptoms and providing information on mental health services, especially those with suicidal thoughts.

This study has some limitations. First, the data were self-reported by the participants. It is possible that the responses did not correspond to actual consulting rates. Second, we could not examine the type of provider from which the respondents received the consultation, because questions about this factor were not included in the survey. There are also some differences between medical institutions, professional consulting institutions, and local health centres. Third, there was no use of psychometric instruments; the data were only based on participant’s subjective view of their depression symptoms. However, the screenings question in the paper were included in the Korean version of the World Health Organization Composite International Diagnostic Interview-Short Form, and validated as a cost-effective screening instrument that could be easily integrated into health surveys [46]. Fourth, people with severe mental illnesses are less likely to respond to a survey. Therefore, it could introduce selection bias in the study population. Finally, the study was based on a cross-sectional survey. Causality could not be confirmed clearly and only association could be confirmed. Despite the above limitations, this study also has several strengths. We used nationally, multistage, stratified collected data that could be considered representative of the Korean population. Second, although most previous studies were conducted in Caucasians, we conducted research on an Asian society. Third, to the best of our knowledge, this study offers new insights into factors associated with the utilization of mental health services. In particular, Korea ranks highest in suicide rates among OECD countries, and the proportion of suicides due to depression is high. Additionally, the government has proposed policies to address depression rates and to recommend mental health consultation for depressive symptoms in Korea. Therefore, our results seem meaningful.

The most common cause of suicide in Korea is psychiatric problems, and the most typical mental illness people suffer is depression. It has been consistently reported that depression is a serious risk factor of suicide attempts and suicide in previous studies [41, 42]. Moreover, mental health services are considered a significant way of preventing suicide [47]. In this study, we examined factors affecting the use of mental health services to treat depressive symptoms and confirmed that educational level is correlated with the utilization of mental health services. The results could have implications for policies on the use of mental health services in Korea.

## Conclusion

This study identified that only 16% of individuals with depressive symptoms obtain mental health consultation and that the rates of use of mental health consultation is lower for older adults men, and the divorced. In particular, educational level shows a significant positive association with mental health consultation in the Korea population. This study also presents information about the vulnerable classes of people receiving mental health consultation services. The government should pay more attention to providing public awareness about the symptoms of depression and increase accessibility of mental health consultation services.

## Abbreviations

CHS: Community Health Survey
CI: Confidence interval
ISCED: International Standard Classification of Education
KSCO: Korean standard classification of occupations
OECD: Organisation for Economic Co-operation and Development
OR: Odds ratio
USA: United States of America
USD: United States dollar
